# Bilateral internal iliac preservation using a short-tip distal branched custom-made device during fenestrated or branched aortic repair

**DOI:** 10.1016/j.jvscit.2026.102485

**Published:** 2026-09-01

**Authors:** Umar Sadat, Elena Trabucco, Ali Ridha, Angelos Karelis, Márton Berczeli, Björn Sonesson, Nuno V. Dias

**Affiliations:** a Department of Clinical Science Malmö, Lund University, Malmö, Sweden; b Vascular Centre, Department of Thoracic and Vascular Surgery, Skåne University Hospital, Malmö, Sweden

**Keywords:** Aortic aneurysm, Aortic dissection, Branched endograft, Internal iliac artery, Endovascular technique

## Abstract

Short common iliac artery anatomy may preclude iliac branch device use during fenestrated or branched aortic repair. We describe two patients treated with a low-profile, short-tip distal branched custom-made device based on an aortouni-iliac configuration and combined with fenestrated or branched repair. The device incorporated iliac side branches to preserve bilateral internal iliac artery perfusion. In aneurysmal anatomy, complete release caused loss of ipsilateral branch access, requiring through-and-through control and balloon-assisted luminal modulation. In chronic dissection, staggered release preserved a working lumen but required adjunctive balloon dilatation. Device configuration, orientation, luminal constraint, and release sequence were critical to technical success.

Short common iliac arteries (CIAs) often preclude iliac branch device (IBD) use. Guidelines support internal iliac artery (IIA) preservation; IBD use requires adequate iliac length, diameter, and morphology for stable deployment and sealing.^1^, ^2^, ^3^ Bilateral IBD implantation may also move the flow divider cranially, increasing the length of the distal reconstruction.

This limitation occurs in aneurysmal degeneration and chronic dissection. In aneurysmal anatomy, the CIA may be short and ectatic. In chronic dissection, limited CIA length may be compounded by true lumen compression, calcification, and restricted maneuverability during fenestrated or branched postdissection thoracoabdominal aortic aneurysm repair.^4^^,^^5^

The innovation is a distal branched custom-made device (CMD) derived from an aortouni-iliac concept. Two contralateral branches above the aortic bifurcation provide separate external iliac artery (EIA) and IIA reconstruction, whereas an ipsilateral IIA branch accompanies the longer CIA/EIA limb. The device therefore functions as a distal aortoiliac reconstruction module rather than a conventional IBD.

The device uses a low-profile, short-tip delivery system (Fig 1, I*A*-*D* and II*A*-*C*) to traverse a deployed fenestrated or branched renovisceral segment while reducing interaction with bridging stents, consistent with the “No Cross” concept.^6^ Branch access depends on controlled release, working-lumen preservation, and rotational orientation.^7^ Sequential catheterization and progressive deployment can preserve working room during branched repair.^8^

**Fig 1:**
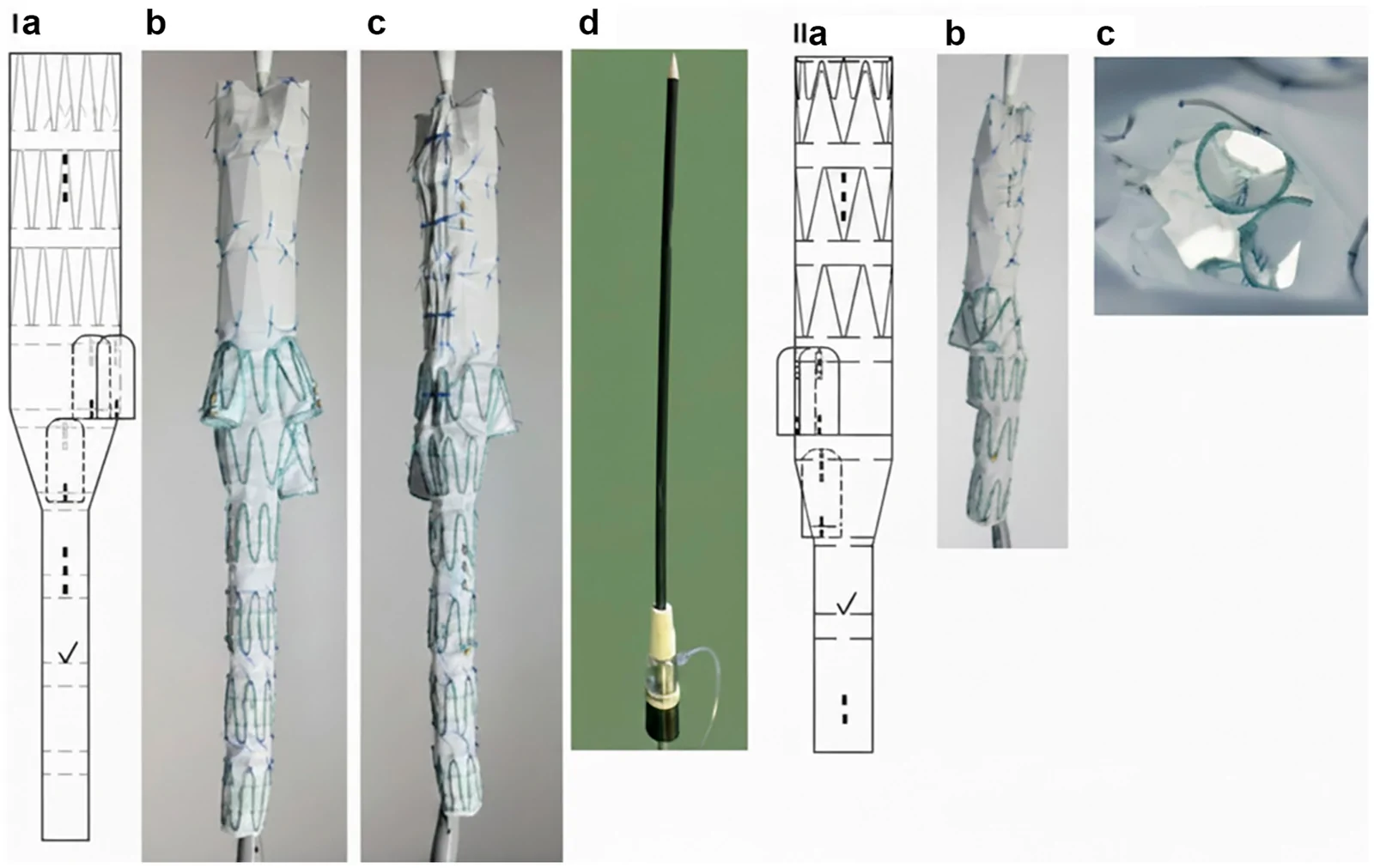
Distal branched custom-made device configurations. Device plans and bench photographs of the two distal branched custom-made devices used for iliac reconstruction in short common iliac artery anatomy. **Ia,** Device plan for the aneurysmal case, demonstrating the asymmetric configuration based on an aortouni-iliac concept; each external side branch measured 8 mm in diameter and 18 mm in length. **Ib** and **c,** Bench photographs showing branch orientation, distal limb configuration, proximal barbs, and diameter-reducing ties. The device was preloaded within a 20F introducer sheath (Flexor; Cook Medical) with a 7.7-mm outer diameter. **Id,** Short-tip delivery system. **IIa,** Device plan for the dissection case, showing the smaller, nonbarbed configuration adapted to constrained true lumen anatomy. The device was preloaded within an 18F introducer sheath (Flexor; Cook Medical) with a 7.1-mm outer diameter. **IIb** and **c,** Bench photographs of the corresponding device, including an endoluminal view of the branch openings.

We describe two patients with short CIAs unsuitable for IBD use who underwent proximal fenestrated or branched repair combined with distal branched reconstruction. Written consent for publication of case details and images was obtained from both patients.

## Technique

Both procedures used fusion imaging, general anesthesia, and percutaneous bilateral femoral and axillary access. All aortic components were patient-specific CMDs manufactured by Cook Medical and were not physician-modified branches. These constructs had no generalized commercial instructions-for-use envelope; sizing, branch geometry, and intended sealing/overlap were individualized from preoperative computed tomography angiography (CTA) with manufacturer engineering review. In these cases, acute branch angles and the need for stable antegrade and through-and-through control made contralateral femoral access impractical.

The first patient underwent Crawford type IV thoracoabdominal aortic aneurysm repair with short, ectatic CIAs (Fig 2, *A*), measuring 25/18 mm right and 36.5/50 mm left (length/diameter); this anatomy was unsuitable for standard bilateral Cook or Gore IBD reconstruction. A patient-specific four-fenestrated preloaded proximal CMD (34 mm proximal diameter, 22 mm distal diameter, and 203 mm length) was introduced from the right femoral approach. An approximately 180° rotational tendency during dilator advancement was managed using the prerotating dilator technique.^7^ Visceral vessels were bridged with balloon-expandable covered stents.

**Fig 2:**
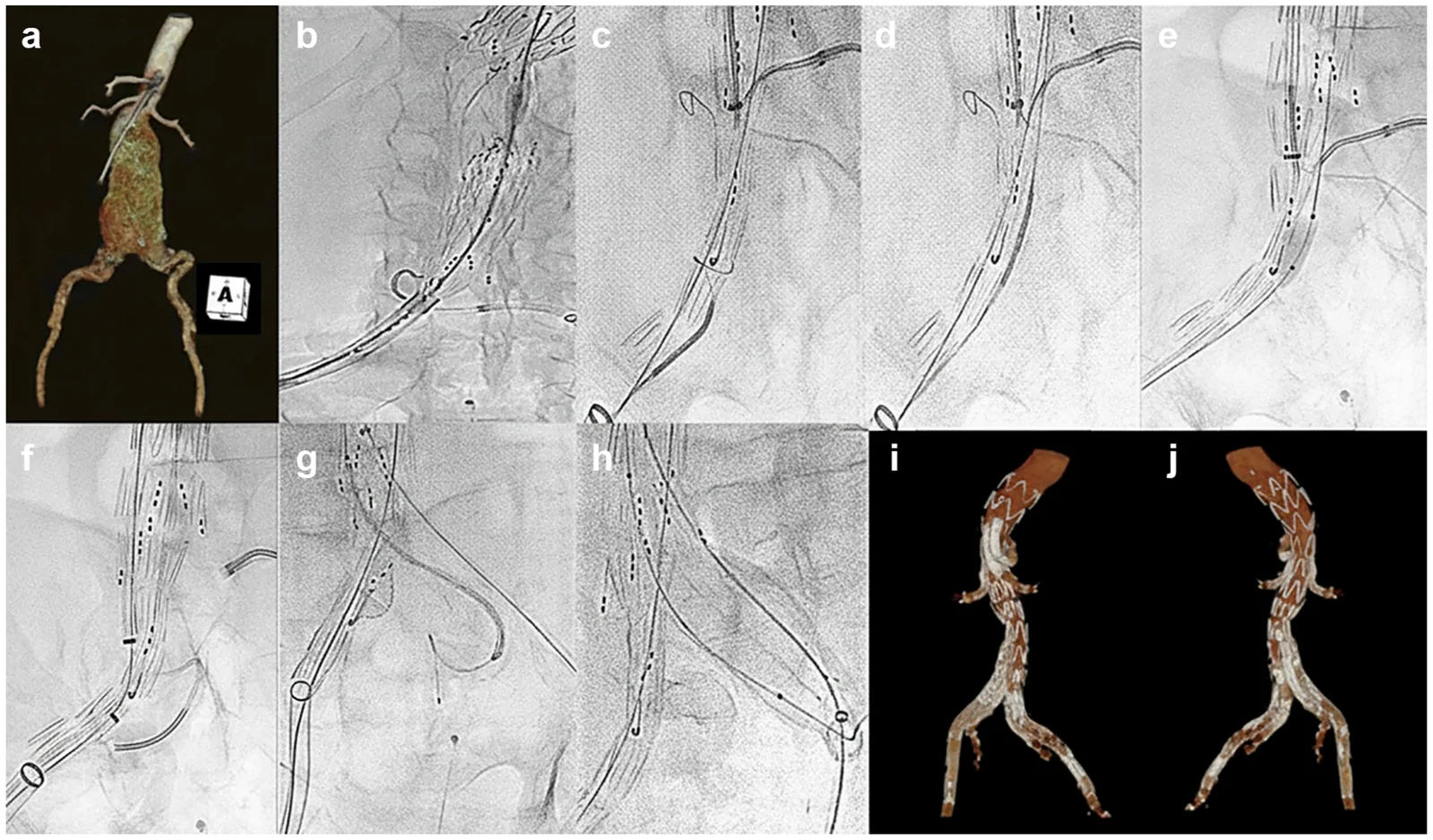
Complete release and access recovery in short, ectatic common iliac artery anatomy. Aneurysmal case. **a,** Preoperative three-dimensional computed tomography angiographic reconstruction showing short, ectatic common iliac arteries unsuitable for standard iliac branch device implantation. **b** and **c,** Complete release of the distal branched component altered the working lumen at the right iliac bifurcation, preventing antegrade access to the ipsilateral internal iliac artery branch. **d** and **e,** Through-and-through wire control and balloon-assisted luminal modulation restored an antegrade access corridor. **f,** Antegrade cannulation of the right internal iliac artery branch. **g** and **h,** Cannulation and reconstruction of the contralateral internal and external iliac artery branches. **i** and **j,** Postoperative computed tomography angiographic reconstructions in anteroposterior and posteroanterior views, respectively, demonstrating patency and preserved configuration of the reconstructed aortoiliac branches.

The distal branched device was a custom graft with a 24 mm proximal diameter, 10 mm distal diameter, and 170 mm length. It was completely opened to restore lower limb flow; staggered release was not used. Complete release into the proximal EIA, combined with iliac tortuosity, impeded antegrade access to the right IIA branch (Fig 2, *B* and *C*). As the graft was planned to seal in the proximal EIA, ipsilateral femoral access was used to advance a wire outside the EIA limb, which was snared from above to establish through-and-through control. Balloon-assisted luminal modulation displaced the graft fabric, restored the working lumen, and enabled antegrade right IIA catheterization (Fig 2, *D-F*). The right IIA was reconstructed with covered stents, and the EIA pathway was relined and reinforced as required.

The contralateral branches opened above the bifurcation and were cannulated antegrade (Fig 2, *G* and *H*). The contralateral IIA and EIA were reconstructed with covered stents, with focal reinforcement where required. Completion angiography confirmed aneurysm exclusion and bilateral IIA perfusion. Postoperative CTA confirmed patency and preserved aortoiliac branch configuration (Fig 2, *I* and *J*).

The second patient underwent branched repair for chronic thoracoabdominal aortic dissection with aneurysmal degeneration, bilateral short calcified CIAs, and compressed true lumen (Fig 3, *A*). The right CIA measured 21 mm in length and 20 mm in diameter. The left CIA measured 26.5 mm in length and 17 mm in diameter; dissection extended 18 mm into the proximal left CIA before transitioning to a nondissected distal CIA measuring 15 mm in diameter. This anatomy was unsuitable for standard bilateral Cook or Gore IBD reconstruction. A patient-specific thoracoabdominal branched CMD with a 36 mm proximal diameter, 18 mm distal diameter, and 158 mm length, incorporating four caudally oriented directional branches, was used proximally. Visceral branches were bridged using the balloon-expandable covered stents.

**Fig 3:**
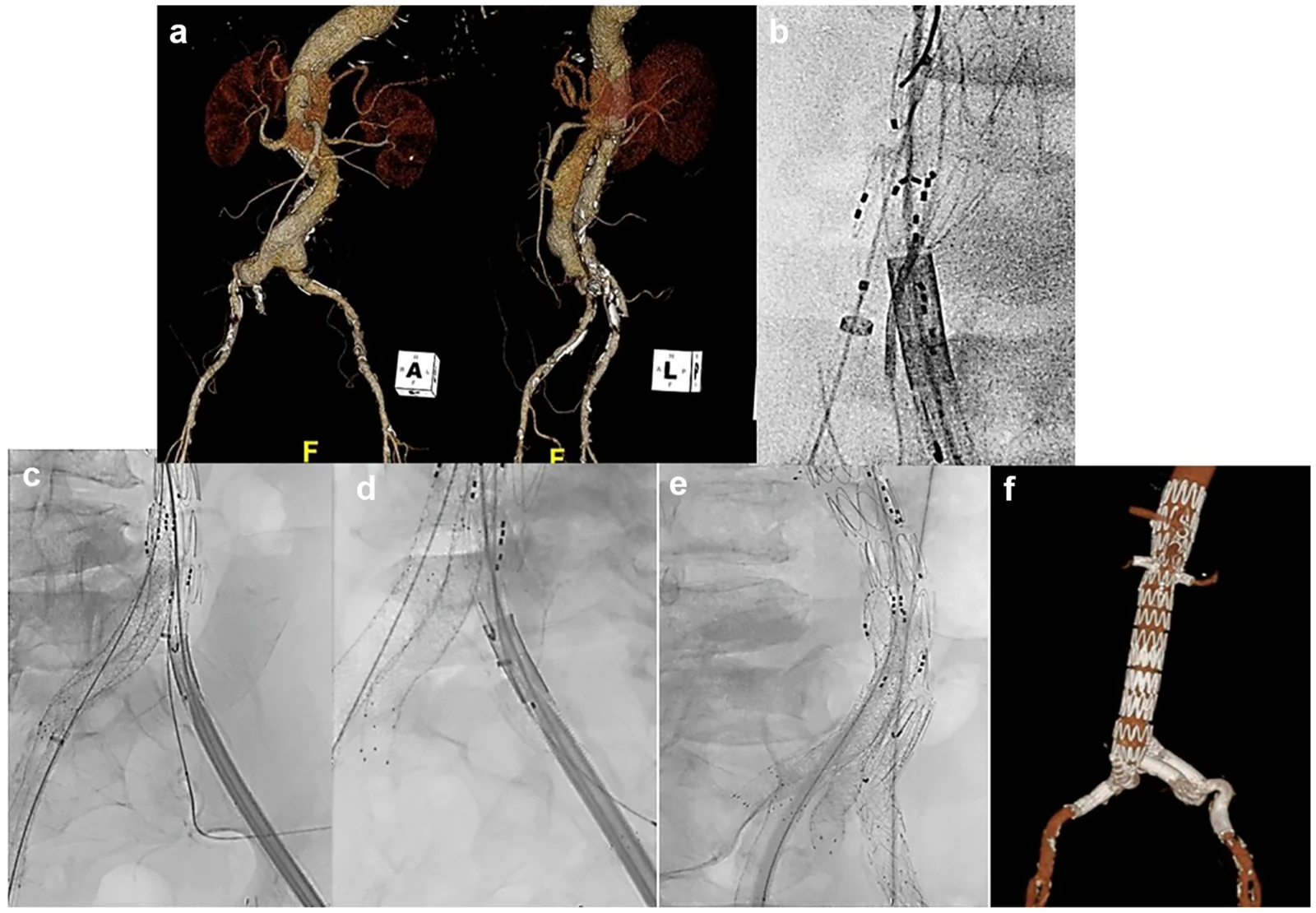
Staggered release in chronic dissection with compressed true lumen. Dissection case. **a,** Preoperative three-dimensional computed tomography angiographic reconstruction showing chronic thoracoabdominal aortic dissection with aneurysmal degeneration, short calcified common iliac arteries, and compressed true lumen. **b,** Staggered opening of the contralateral branches while the remaining distal component remained constrained. **c** and **d,** Cannulation and reconstruction of the ipsilateral left internal iliac artery within the constrained true lumen. **e,** Completion fluoroscopic image demonstrating the integrity and configuration of the implanted stents. **f,** Postoperative three-dimensional computed tomography angiographic reconstruction in an anteroposterior view, showing the completed aortoiliac reconstruction with patent bilateral internal iliac artery branches.

The distal branched device was a custom graft with a 20 mm proximal diameter, 12 mm distal diameter, and 134 mm length, introduced from the left femoral approach. The distal aortic and left CIA true lumen ranged from approximately 18 to 26 mm. Staggered release followed sequential catheterization and progressive deployment principles.^8^ The graft was partially unsheathed to expose the contralateral EIA and IIA branches, whereas the distal component remained constrained (Fig 3, *B*). The EIA was bridged from femoral access and the IIA from axillary access. Balloon-expandable reinforcement was added where competing stent trajectories occupied the constrained true lumen. The main endograft was then unsheathed beyond the left IIA branch, permitting ipsilateral IIA reconstruction (Fig 3, *C* and *D*); balloon dilatation was required to maintain usable space.

The remaining iliac branches were reconstructed with covered stents and selective reinforcement according to trajectory and radial-support requirements.

Completion fluoroscopy confirmed the final stent configuration (Fig 3, *E*). Postoperative CTA demonstrated patent bilateral IIA reconstructions and preserved aortoiliac configuration (Fig 3, *F*). Follow-up at 1 month for the aneurysmal case and 3 years for the dissection case confirmed branch patency without type I/III endoleak or structural failure.

## Discussion

Short CIA anatomy remains a key limitation to IBD use. Distal branched reconstruction can preserve IIA perfusion, but success depends on device architecture, luminal environment, release sequence, and orientation.

The aneurysmal case demonstrates the risk of complete release without staggered constraint. Expansion of the long CIA/EIA limb and IIA branch eliminated the working lumen required for antegrade IIA catheterization. Through-and-through access and balloon-assisted luminal modulation restored access, illustrating how unrestricted expansion can remove the pathway needed to reach a target branch.

The dissection case illustrates the opposite problem. The true lumen was narrow, calcified, and constrained. Staggered release exposed the contralateral branches and later the ipsilateral IIA branch, whereas the remaining component stayed constrained. This preserved a working lumen, although balloon dilatation was still required, consistent with sequential catheterization and progressive deployment principles.^8^ Septal modification is an alternative when severe chronic-dissection constraint prevents adequate working space.^9^

Complex fenestrated/branched repair is inherently modular.^10^ Commercial IBDs are geometry-dependent.^3^ The bifurcated-bifurcated-IBD treats CIAs as short as 2 to 2.5 cm but provides one IIA branch; bilateral reconstruction requires another IBD.^11^ Manesh et al^12^ incorporated both IIAs using physician-modified branches. Access to manufacturer CMD programs varies by jurisdiction. Our CMD instead consolidates bilateral IIA/EIA reconstruction in one asymmetric aortouni-iliac-derived module delivered by a low-profile short-tip system. Geometry, sealing/overlap, component interaction, and durability were reviewed with manufacturer engineers.

Bridging-stent selection was anatomy-driven. Self-expanding covered stents provided conformability across curved iliac trajectories, whereas balloon-expandable components provided precision and focal radial support where adjacent stents competed within the constrained lumen.^13^

These cases are limited by their small number and custom design; long-term durability cannot be established. Controlled graft release, preservation of luminal working space, and accurate orientation may facilitate distal branched reconstruction when standard IBDs are unsuitable.

## Funding

No external funding was received for this study.

## Patient consent

Written consent for publication of case details and images was obtained from both patients.

## Declaration of generative AI and AI-assisted technologies in the writing process

During the preparation of this work, the authors used ChatGPT to improve readability and language. After using this tool, the authors reviewed and edited the content as needed and take full responsibility for the content of the publication.

## Disclosures

A.K. has served on a speakers’ bureau for W. L. Gore & Associates. N.V.D. reports intellectual property and advisory/speaker roles with Cook Medical, advisory/speaker roles with W. L. Gore & Associates, and an advisory role with Artivion Inc. The remaining authors declare no conflicts of interest.
